# Multiprofessional committee on auditory health – COMUSA

**DOI:** 10.1590/S1808-86942010000100020

**Published:** 2015-10-17

**Authors:** Doris Ruthy Lewis, Silvio Antonio Monteiro Marone, Beatriz C.A. Mendes, Oswaldo Laercio Mendonça Cruz, Manoel de Nóbrega

**Affiliations:** 1 Full professor, PUCSP Phonoaudiology School, graduate course, Speech therapist at the Centro Audição na Criança – DERDIC/PUCSP; 2 Associate professor of otorhinolaryngology. medical school, USP, Full professor of otorhinolaryngology, medical school, PUC de Campinas; 3 Associate professor, Phonoaudiology School, PUCSP, Speech therapist at the Centro Audição da Criança – DERDIC/PUCSP; 4 Associate professor of otorhinolaryngology, medical school, USP, Affiliated professor of otorhinolaryngology, medical school UNIFESP; 5 Doctorate in otorhinolaryngology, Unifesp

**Keywords:** hearing loss, hearing tests, newborn hearing screening

## Abstract

Created in 2007, COMUSA is a multiprofessional committee comprising speech therapy, otology, otorhinolaryngology and pediatrics with the aim of debating and countersigning auditory health actions for neonatal, lactating, preschool and school children, adolescents, adults and elderly persons. COMUSA includes representatives of the Brazilian Audiology Academy (Academia Brasileira de Audiologia or ABA), the Brazilian Otorhinolaryngology and Cervicofacial Surgery Association (Associação Brasileira de Otorrinolaringologia e Cirurgia Cérvico Facial or ABORL), the Brazilian Phonoaudiology Society (Sociedade Brasileira de Fonoaudiologia or SBFa), the Brazilian Otology Society (Sociedade Brasileira de Otologia or SBO), and the Brazilian Pediatrics Society (Sociedade Brasileira de Pediatria or SBP).

## NEONATAL AUDITORY HEALTH

### Universal neonatal auditory screening – UNAS

Because more hospital units are implementing universal neonatal auditory screening programs (UNAS), because state and city laws are being approved, because questions have been sent to entities by their associate members, and because of federal and state funded research in neonatal auditory health, the abovementioned entities have decided to publish advice on UNAS as a guideline for professionals.

## INTRODUCTION AND BACKGROUND

Hearing develops in increasingly complex stages from intrauterine life onwards. For a child to acquire language and develop speech, he or she should be able to detect, locate, discriminate, memorize, recognize, and finally understand sounds.^1,^^2,^^3,^^4^ Any, and especially the initial of these steps, are extremely important for the whole process to take place;^5^,^6^, any interruption will result in significant loss of function during child development. Thus, measures should be undertaken promptly to minimize eventual difficulties doe to sensory deprivation. Therefore, neonates with hearing loss should be identified within the first month of life, even if the clinical history reveals no indicators of risk for the probability of hearing loss.^2,^^3,^^6,^^7,^^35^

The central nervous system is very plastic when stimulated early, before 12 months of age, which increases the number of nerve connections and improves the results of auditory rehabilitation and language development in children with hearing loss.^6,^^8,^^9,^^10^ The first six months are decisive for future development; for this reason, speech therapists, otorhinolaryngologists and pediatricians have been concerned with promoting awareness campaigns for the population and for healthcare professionals about the importance of early identification and diagnosis of hearing loss and prompt medical and phonoaudiological interventions.^6,^^9,^^10,^^11,^^12,^^13,^^14^

There are several causes of congenital hearing loss, which is acquired prenatally of within the first days following birth. Genetic recessive inherited auditory system damage, which causes hearing loss not associated with any syndrome, is the most frequent cause of hearing loss reported in developed countries; it generally is expressed early, before language develops. Other than heredity, the most common causes of congenital hearing loss are due to very low birth weight (less than 1,500 g), hyperbilirubinemia, congenital infection such as rubella, toxoplasmosis, cytomegalovirus, syphilis, and neonatal use of ototoxic drugs. Children with head and neck malformations and and/or syndromes that include hearing loss may also be affected. It is common for such hearing loss risk factors to be associated among neonates, especially if they remain in neonatal ICUs for over five days.

It is not uncommon for the cause of hearing loss to remain unclear; hearing loss is idiopathic is about half of neonates. As such, selective neonatal auditory screening done only in children selected for hearing loss risk factors identifies about 50% of hearing loss patients.^7,^^11,^^35^ Therefore, universal screening is the ideal method, and has been defended by all professionals acting in this area. UNAS is here understood as performing auditory tests in over 95% of neonates, preferably before being discharged from hospital.^7^

In developed countries, sensorineural hearing loss affects one in every 1,000 newborns; 40% are due to hereditary factors, 30% are due to a variety of acquired conditions, and 20% are of unknown etiology.^15^ There have been few population base studies of neonates in Brazil. A study in a private hospital in Sao Paulo from 1996 to 1999 screening 4,631 neonates (90.6% of live births) found 10 children with permanent hearing loss (0.24%), a prevalence of 2.4/1000.^16^

A study of 200 children and adolescents by the Sao Paulo Federal University (UFSP) in 1998 revealed that diagnostic confirmation of hearing loss within the optimal neuronal plasticity phase – up to age 2 years – was made in only 13% of cases, even though parents had suspected hearing loss in 56% of cases in this age group. Time wasted – over two years – between suspected and confirmed hearing loss occurred in 42% of cases, underlining the need for rethinking our health care model.^17^ Another study in 2005 at the same institution, involving 519 children and adolescents revealed that the main causes of hearing loss were: idiopathic (36.6%); genetic (13.6%), including 4.0% of consanguinity cases; congenital rubella (12.9%); perinatal causes (11.4%); meningitis (10.6%); and other causes (14.9%).^18^

Early functional and etiological diagnosis of hearing loss and prompt medical, phonoaudiological or surgical interventions minimize the effects of hearing loss in children.^10^ Developments in neonatal auditory screening and identification methods since the 1990s have led to greater efforts for diagnosing and initiating rehabilitation before six months of age.

Guidelines published in the US by the National Institutes of Health^19^ in 1993 and by the Joint Committee on infant Hearing in 1994^20^ have led to a gradual and continuous implementation of UNAS throughout North-American states. In 2006, 95.7% of newborns in 50 states were assessed before being discharged from hospital.^21^ Also in the US, in 1999 the American Academy of Pediatrics recommended UNAS with quality metrics to be reached. Such indicators refer to carrying out neonatal auditory screening in at least 95% of live newborns and identifying hearing loss of at least 35dB in the best ear; they also suggest that there should be no more than 4% false positives and that the rate of false negatives should be zero.^22^ The Joint Committee on Infant Hearing also underscored these guidelines in 2000.^23^

UNAS was recommended in Europe in 1998 in a consensus signed by several countries at a scientific event for this purpose. Below are the justifications for implementing UNAS:^24^

• Permanent hearing loss is a major public health issue; satisfactory results may be attained with treatment if interventions take place within the first months of life;
• Effective methods are available in clinical practice to identify hearing loss; these methods include recording evoked otoacoustic emissions (EOAE) and the brainstem auditory evoked potential (BAEP);
• Neonatal auditory screening only of newborn babies at risk of hearing loss has been suggested as a lower cost strategy; it may, however, not identify about 40–50% of children with permanent hearing loss;
• UNAS is only the first step of a neonatal auditory health program; diagnostic and rehabilitation measures should ensue.

In Brazil, the first multiprofessional guidelines on auditory health in children were published in 1995, the result of a workgroup that was organized in the X International Audiology Meeting (X Encontro Internacional de Audiologia). The debate forum on “Hearing in Children” made recommendations for auditory healthcare in children, focusing on joint and multiprofessional actions in the public and private spheres to promote and protect auditory health. This document suggests early identification, diagnosis and rehabilitation according to the principles of integral healthcare for children.^25^

The Universal Neonatal Auditory Screening Support Group (GATANU) was created in Brazil in 1998; its aim was to disseminate and sensitize society to the need for an early diagnosis of deafness.^26^.

In 1999, the Brazilian Committee on Hearing Loss in Infancy (CBPAI), composed of speech therapists, otorhinolaryngologists and pediatricians, published guidelines on the need for identifying hearing loss in neonates and implementing the UNAS, preferably before the children were discharged from hospital. Auditory screening was recommended within three months of age for those born at home.^27^

In 2000, the Federal Board of Phonoaudiology published the Recommendation CFFa. n^o^ 05/00, which suggested implementing auditory screening in neonates using objective methods published in medical literature, such as evoked otoacoustic emissions (EOAE) and the brainstem auditory evoked potential (BAEP). The recommendation also points out the required steps and the need for multiprofessional teams if a further diagnosis is required.^28^

Thus one can state that Brazil, for over the past 10 years, has carried out actions to underline the importance of identifying and treating hearing loss at an early age.

Several city and state laws making UNAS compulsory in maternity hospitals have been passed in Brazil. However, it is clear that few public maternity hospitals have a systematic UNAS program.^29^

By screening we mean a simple, quick and low cost process to identify children at a higher probability of having altered auditory function. Children that do not pass the screening tests should be referred for more complex diagnostic procedures.^30^

The following principles should be applied during screening procedures: the prevalence of the disorder being tested should justify universal screening; screening procedures should be sensitive and specific; diagnostic and treatment resources should be available; cost should be compatible with the desired effectiveness; this actions should be accepted by healthcare professionals and the population.^31^

The most recent international publication containing guidelines for UNAS was published in 2007 by the Joint Committee On Infant Hearing;^32^ it lists eight principles to provide an effective basis for early diagnostic and therapeutic systems:

1 All neonates should have priority access to UNAS with physiological measures within the first month of life;
2 All children with unsatisfactory test and retest UNAS results should have access to diagnostic measures to confirm hearing loss within three months of life;
3 All children with confirmed permanent hearing loss should start treatment and intervention measures after diagnosis within six months of life;
4 Interventions should include family and caretaker participation, respecting the rights of child and family, confidentiality, and the current ethical and legal requirements;
5 Children and their families should have prompt access to high quality and high complexity technology, such as sound amplification hearing aids and cochlear implants, as well as other support devices, whenever applicable;
6 All normal hearing children should have their hearing, language development and communication ability monitored, even in the absence of hearing loss risk factors, by duly trained professionals:
7 Children with permanent hearing loss and their families should be followed-up by experienced interdisciplinary teams in the care of children with hearing loss:
8 Computer database systems should be generated to monitor the results and effectiveness of identification, diagnosis and early intervention measures for hearing loss in neonates.

The Joint Committee on Infant Hearing^32^ recommends using physiological procedures (EOAE and BAEP); for neonates with no hearing loss risk factors, any one of these methods is considered as appropriate. If BAEP testing is used in this population, it may be possible to identify neural auditory disorders, such as the auditory neuropathy spectrum, as well as cochlear hearing loss.^33^

The Joint Committee on Infant Hearing (2007)^32^ comments the fact that some unit use EOAE recordings as initial screening, followed by BAEP testing if the former method fails, to decrease false positive rates and the need for future monitoring. Some caveats in that paper deserve attention: 1. Children that do not pass EOAE testing, and that present normal BAEP recordings, may eventually show mild losses (from 25 to 40 dB). 2. Care should be taken with adequate EOAE test results and failed BAEP recordings, as this finding suggests the auditory neuropathy spectrum.

For neonates with hearing loss risk factors, especially those admitted to neonatal ICUs, the Joint Committee on Infant Hearing^32^ recommends BAEP testing for screening purposes as the only appropriate method for this group, due to an increased occurrence of the auditory neuropathy spectrum. If auditory screening detects findings in children with hearing loss risk factors, prompt referral to an experienced pediatric medical team for retesting and diagnosis.^32^

Professionals who implement, coordinate or work, teach and research the UNAS are encouraged to read the Joint Committee on Infant Hearing (2007)^32^ guidelines.

The US Preventive Services Task Force published similar guidelines in July 2008 for carrying our UNAS with EOAE or BAEP-A testing to identify 30–40 dB hearing losses; in this case, the process may include two steps (EOAE followed by BAEP-A) to improve sensitivity (0.92) and specificity (0.98). Auditory screening in neonates should be done on the first day of life, preferably before the newborn is discharged from hospital. This particular North-American publication describes studies showing the strong contribution of UNAS for early diagnosis and intervention and the reliability of objective tests. Further publications on studies demonstrating the results for language development in children identified by applying the UNAS.^35^,^42^

Recent published studies have attempted answer language developmental issues in children with identified, diagnosed and treated conditions within the first months of life; it appears that such evidence is being demonstrated.^41^

Authors who have shown that this population group had a higher rate of retrocochlear hearing loss – not identifiable with EOAE recordings – have suggested using BAEP-A in the UNAS of higher risk children. The auditory neuropathy spectrum falls within this situation. Thus, low birth weight children, which generally present other associated risk factors, and those admitted to neonatal ICUs, may require BAEP-A in the UNAS, because of increased synergy among risk indicators. Studies have shown that hearing needs to be monitored in these children because of the possibility of late onset hearing loss or the progression of hearing losses diagnosed at this initial point.^36^,^40^

The techniques and protocols are being described to reduce the number of false positives in UNAS. Use of EOAE and BAEP-A recording in different steps has shown a positive effect. Studies have shown that using EOAE as a first step, followed by BAEP-A as a second step, significantly decreases the number of neonates referred for diagnosis when there is initial failure of hearing.^38^,^39^

Although genetic congenital hearing losses are important, particularly those involving Conexin^26^, several unresolved technical and ethical issues lack evidence, so that neonatal screening for GJB2 is still not included. Genetic testing and counseling is recommended after hearing loss is confirmed. So, studies are still needed before implementing neonatal screening for GJB2 mutations.^37^

The epidemiology of hearing loss in children in Brazil requires further study in order to put in effect preventive measures. There are few data on the determining factors and prevalence of hearing loss in neonates. UNAS may help provide prompt interventions in children with hearing loss and foster appropriate public policies for this age group.

The National Policy for Auditory Healthcare is available in most of the country; there are 127 auditory health reference centers, of which 80 are high complexity units able to care for neonates with specialized diagnostic needs and to select and provide sound amplification hearing aids and speech therapy. Maternity hospitals with auditory health reference units for diagnosis and medical and phonoaudiological interventions may already implement neonatal auditory screening.^34^

## COMUSA RECOMMENDATIONS

After analyzing the literature of data showing evidence on the identification, diagnosis and early intervention in neonates and lactating children with hearing loss, COMUSA recommends:

1 A neonatal auditory health program with all prevention, diagnosis and hearing loss rehabilitation measures should be implemented, as follows:
  • Promote the health of pregnant women, neonates and lactating children;
  • Prevent hearing loss by applying specific measures to be put in place following epidemiological prevalence and determinant factor studies on hearing loss in neonates;
  • Identify hearing loss by auditory screening with sensitive and specific methods; electrophysiological measures are recommended (brainstem auditory evoked potentials – BAEP) and/or electroacoustic methods (recording evoked otoacoustic emissions – EOAE – by transient or distortion product stimuli);
  • Make available referral for medical and audiological diagnosis and laboratory tests, as needed; imaging and genetic evaluation may be required;
  • Make available medical treatment and speech therapy, including the selection and indication of individual sound amplification hearing aids, cochlear implants, and family support, as needed;
  • Make available high quality technology, assuring adequate treatment for a good development of hearing, language acquisition, and consequently, educational and social development in children with hearing loss;
  • Medical care and speech therapy should respect the needs and choices of parents or caretakers of neonates, offering different scientifically and technologically supported approaches and methods.
2 Implanting a universal neonatal auditory screening program (UNAS), for all neonates preferably before being discharged from hospital.
3 For home births, out of hospital environments, or in maternity hospitals that are still implementing universal auditory screening, tests should be one within the first month of life.
4 It is suggested that strategies for implementing UNAS in all maternity hospitals be written. Meanwhile, sites for carrying out auditory screening within the first months should be defined.
5 Implementation of a UNAS program may be gradual in order to better organize the necessary actions. The goal for implementing auditory screening universally, however, is three years.
6 It is suggested that a dissemination/information plan about this action for neonates be written during implementation of neonatal auditory screening, for both healthcare professionals and the general population, so that the importance of UNAS and its methods be widely known among the population.
7 It is suggested that every hospital with a neonatal auditory screening program should elect a maternity hospital coordinator for this action, such as a neona-tologist, or otorhinolaryngologist or speech therapist with proven experience or specialization in audiology. In smaller municipalities or hospitals that are unable to elect such a coordinator, it is recommended that implementation of a neonatal auditory screening program be carried by a healthcare professional supervised and supported by a coordinator from another hospital with experience in implementing UNAS. Scientific societies that undersign this document are ready to provide further information to their associate members about implementing a UNAS, helping supervise and legitimatize these programs.
8 It is suggested that parents/caretakers receive explanations about the importance of UNAS, and that they be asked to sign a free informed consent form for the test, or a term of responsibility if they refuse the test. Furthermore, it is suggested that parents/caretakers sign upon receiving the results of tests and orientation before the neonate is discharged from hospital, for both normal and altered tests. All test results (test and retest) should be provided in printed form to parents/ caretakers.
9 For neonates with no indicated risk of hearing loss, the recommended method for UNAS is the transient or distortion product stimuli evoked otoacoustic emissions (EOAE) test. Its purpose is to identify cochlear hearing loss up to 35 dBHL. It is a low cost easily applied test, added to which this neonate population group has a low prevalence of auditory neuropathies.
10 Because of false negatives due to vernix in the outer ear in the first days of life, a second evaluation is recommended within 30 days of hospital discharge in all cases of absent (altered) EOAE, even if in one ear only. At this visit, both ears should be retested, even in test failure occurred in only one ear during the first test.
11 Cases where EOAE testing fails should undergo automatic BAEP (BAEP-A) before being discharged from hospital and/or when being retested. This measure aims to reduce the number of unnecessary referrals for diagnosis. Normal BAEP-A responses in both ears should be considered as satisfactory screening results. However, parents or caretakers should be informed that if hearing difficulties arise or are suspected, an auditory healthcare reference unit should be sought immediately.
12 If within 30 days screening tests fail, the patient should immediately be referred for a full otorhinolaryngological medical and phonoaudiological diagnosis. A diagnostic team consisting of otorhinolaryngologists and speech therapists should have experience in evaluating neonates and lactating children.
13 For neonates with normal tests, orientation on the care of auditory health should be given, as well as a table for monitoring the development of hearing abilities and language, so that these may be observed within the family.
14 A copy of the test and relevant annotations should be annexed to the child's records, including the data and signature of the healthcare professional in charge of the UNAS process, in the case of normal or altered tests, and with their referrals, if applicable.
15 For neonates with risk indicators of hearing loss, BAEP-A testing at 35 dBHL is recommended as the initial screening method. This method makes it possible to diagnose cochlear, retrocochlear, and sensorineural conditions, including the auditory neuropathy spectrum, which are more prevalent in the higher risk population. EOAE testing may be associated with BAEP-A, given the risk of more unsatisfactory results because of middle ear changes, but not because of permanent sensory hearing loss.
16 If BAEP-A recording are absent in the initial test of neonates at risk for hearing loss, it is recommended that these patients be referred to diagnostic units immediately, without retesting, as described for other neonates.
17 All test results should be given to parents or caretakers, and annotated in the child's health report and hospital records; parents or caretakers should sign an appropriate form or terms upon receiving the referrals, results and orientation.
18 For neonates with risk indicators of hearing loss and satisfactory BAEP-A testing during screening, auditory function should be monitored by a trained professional until the third year of life.
19 The main hearing loss risk factors described in the specialized literature included any of the following factors, and are therefore listed in these guidelines:^6,^^10,^^17,^^18,^^23,^^32^
  • Parental concern with child development with regards to hearing, speech or language;
  • History of permanent deafness cases in the family starting during infancy, and therefore considered a hereditary risk. Consanguinity cases should be included here;
  • ICU stay for over five days, or the occurrence of any of the following conditions, regardless of the duration of ICU stay: extracorporeal ventilation, assisted ventilation; exposure to ototoxic drugs such as aminoglicoside antibiotics and/or loop diuretics; hyperbilirubinemia; severe perinatal anoxia; neonatal Apgar score from 0 to 4 in the first minute or 0 to 6 in the fifth minute; birth weight below 1,500 grams; preterm birth, or low weight for the gestational age;
  • Congenital infections (toxoplasmosis, rubella, cytomegalovirus, herpes, syphilis, HIV infection);
  • Craniofacial anomalies involving the ear and temporal bone.
  • Genetic syndromes that usually express hearing loss (Wardenburg, Alport, Pendred, among others);
  • Neurodegenerative disorders (Friedreich's ataxia, Charcot-Marie-Tooth syndrome);
  • Postnatal bacterial or viral infections such as cytomegalovirus infection, herpes, measles, smallpox, and meningitis;
  • Cranial trauma;
  • Chemotherapy.
20 It is suggested that a computerized database on auditory neonatal auditory screening be made so that the coordinator may monitor the results monthly, and as a tracking tool for cases that may be lost to follow-up, or that did not conclude all the necessary retest or diagnostic steps. Software is available, and other computer programs may be written by the information technology teams of states and municipalities.
21 Monthly, quarterly and annual reports should be made to help monitor the development of the neonatal auditory screening program, quality control, and tracking information. The same quality control metrics used in the auditory screening program of the Joint Committee on Infant Hearing (2007) are recommended:
  • Screening indices over 95% of live births, with attempts to reach 100% of live births;
  • Screening tests should be carried out within the first month of life;
  • A less than 4% rate of neonates referred for diagnosis;
  • Quality indicators in the diagnosis phase are the visit rates upon referral for diagnosis; 90% of referred neonates should be present and the diagnostic visit, and should have been diagnosed by three months of life;
  • It is suggested that 95% of lactating babies with confirmed permanent bilateral hearing loss should initiate use of sound amplification within one month of the diagnosis.

COMUSA DOES NOT RECOMMEND including genetic screening to identify hearing loss-related genetic abnormalities at this point in time, because of the need to investigate the cost, effectiveness and feasibility of genetic studies in maternity hospitals.

Implementing a UNAS program is only meaningful if after its completion a correct diagnosis is made and auditory rehabilitation processes and language development are implemented in sequence.

**Figure 1:**
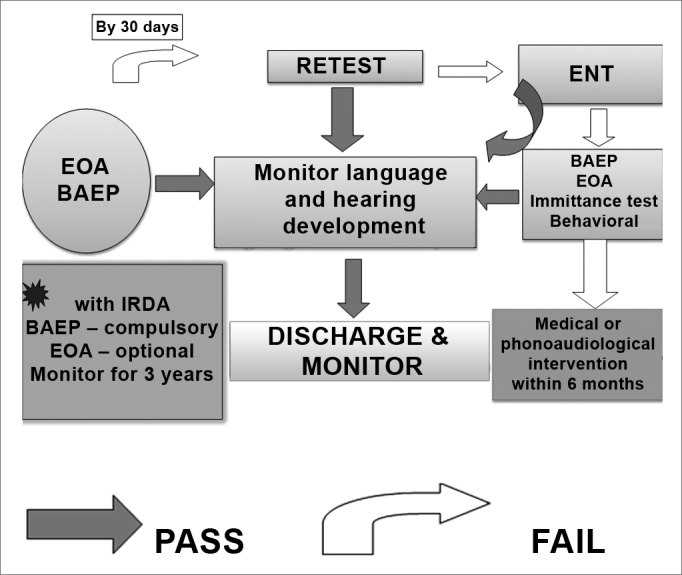
UNAS algorithm
